# Molecular detection and characterization of *Anaplasma marginale* and* Babesia canis vogeli* infecting dogs in Luxor, Egypt

**DOI:** 10.1038/s41598-024-67009-6

**Published:** 2024-07-23

**Authors:** Hassan Y. A. H. Mahmoud, Moshera S. Shahat, Ragab M. Fereig, Alsagher O. Ali, Walaa F. A. Emeish, Ahmed M. Soliman, Fatma A. Khalifa, Tetsuya Tanaka

**Affiliations:** 1 Division of Infectious Diseases, Animal Medicine Department, Faculty of Veterinary Medicine, South Valley University, Qena, 83523 Egypt; 2 Division of Internal Medicine, Animal Medicine Department, Faculty of Veterinary Medicine, South Valley University, Qena, 83523 Egypt; 3 Department of Fish Diseases and Management, Faculty of Veterinary Medicine, South Valley University, Qena, 83523 Egypt; 4 Biotechnology Department, Animal Health Research Institute, Agricultural Research Center, Dokki, 12618 Giza Egypt; 5 Laboratory of Infectious Diseases, Joint Faculty of Veterinary Medicine, Kagoshima University, Kagoshima, 890-0065 Japan; 6 Laboratory of Animal Microbiology, Graduate School of Agricultural Science, Tohoku University, Sendai, 980-8572, Japan

**Keywords:** *Anaplasma marginale*, *Babesia canis vogeli*, Dog, Egypt, Infectious-disease diagnostics, Parasite biology

## Abstract

Tick-borne diseases in animals are increasing rapidly worldwide, but there is insufficient information about tick-borne diseases infecting dogs in southern Egypt. Thus, in the current study, we detected the presence of *Anaplasma marginale (A. marginale)* and *Babesia canis vogeli* (*B. canis vogeli*) in the blood of dogs. The results revealed that 4/100 (4%) were positive, and a higher infection rate was found in males (75%), than females (25%). The phylogenetic analysis for the *major surface protein 4* (*msp4*) gene in this study was compared with amplicons separate from other reported isolates with alignment by identity 100% with cattle and camels from Egypt, and the phylogenetic analysis for the *B. canis vogeli small subunit ribosomal RNA* (*SSU rRNA*) gene in this study identified identity by 99.89% with dogs from Egypt. This report is considered the first report in southern Egypt about *A. marginale* in dogs based on the sequence analysis of the *msp4* gene, providing new data for the classification and identification of *A. marginale* in dogs compared to *A. marginale* isolated from other animals in southern Egypt.

## Introduction

In recent decades, several new, emerging, and reemerging tick-borne rickettsial diseases have been recognized as a significant public health concern worldwide^1,2^. Similarly, tick-borne diseases cause significant financial losses in the production of food animals worldwide^3^. Viral, bacterial, and protozoan diseases transmitted by vectors are often prevalent in tropical and subtropical regions^4^, including the Middle East and North Africa. The development of vector-borne canine pathogens, including *Ehrlichia* spp., *Borrelia* spp., and *Dirofilaria* spp. transmitted by ticks is of great importance for dogs^5,6^.

Dogs are sub clinically infected with tick-borne pathogens and serve as reservoirs for their owners and contact animals^7,8^. Tick-borne disease symptoms are typically diffuse and overlapping, especially in mixed infections, therefore accurate diagnostic methods are required for an effective treatment and control strategy^9^. Tick-borne pathogens are typically diagnosed through the microscopic examination of blood smears, but these methods are less sensitive and require highly skilled investigators. Traditional techniques are used to detect tick-borne pathogens in peripheral blood and tick types with lower sensitivity and specificity than molecular methods. Molecular techniques have been used to confirm the identity of canine blood parasites. Furthermore, the studies including molecular analysis studies aided in a better understanding of new dog-infecting species^10^.

Stray dogs are usually maintained in close contact with humans while roaming freely without significant human care. Such dogs frequently have parasitic infections and infestations, which serve as reservoirs for various zoonotic parasites that pose potential hazards to public health. The data on the prevalence and diversity of parasites in dogs is limited, particularly in developing and low-income countries^11^.

The protozoan morphology (piroplasm merozoites) within the red blood cell has traditionally been used as the primary taxonomic determinant based on a microscopic examination of a blood smear, this determination can categorize these protozoa as large size *Babesia* species (*B. canis*) or small size *Babesia* species (*B. gibsoni*), following that, molecular techniques identified several *Babesia* species that can infect dogs^12^.

The life cycle of *Babesia* protozoans spends part of it in the tick vector, but the merozoites circulating in the blood of infected animals can be transmitted directly to a healthy host via blood transfusion^13^. The first clinical evidence of possible vertical transmission of *B. canis*^14^ and *B. microti-*like spp*.* has also been documented^15^. *Babesia*-infected dogs may be asymptomatic^16^ or exhibit various clinical signs ranging from mild to severe and fatal. Clinical manifestations include anorexia, lethargy, fever, pale mucous membrane, jaundice, and renal disease depending on the parasite and the host's age, sex, and breed^17–19^. Subclinical signs of *B. vogeli* infection have been observed in both natural and experimental in vivo infections. The pathogen causes severe acute infection in immunocompromised animals, such as splenectomized dogs. The clinical signs can be accompanied by fever, anorexia, malaise, regenerative anemia, thrombocytopenia, and an increased white blood cell count^20^.

*Anaplasma marginale* is a frequently mechanically transmitted pathogen to susceptible cattle by blood-contaminated mouthparts of the blood sucking Diptera (*Tabanus* and *Stomoxys)* or via fomites^21^. The only site of replication in cattle is within the erythrocytes, where it forms membrane-bound vacuoles containing 4–8 *A. marginale*^22^. The prepatent period ranges from 7 to 60 days (depending on the infective dose), then up to 70% of erythrocytes may become infected during acute infections and with clinical signs^23^. Bovines older than one year are the most vulnerable to developing clinical diseases, severe anemia, icterus (without hemoglobinuria), fever, weight loss, lethargy, depression, and abortion were the main clinical signs observed^24^. The postmortem findings included icterus, splenomegaly, hepatomegaly, and petechial hemorrhage on the heart and pericardium. The blood was thin and watery, and all tissues were pale^25^, cattle that survived acute infections may remain carriers for the rest of their lives^3^. This study is part of many studies aimed to examine the anaplasmataceae and piroplasmosis in the different animal species including camel, cattle, buffalo, sheep, and goat in southern Egypt, the varied in breed, age, and gender, with most of animals showing no signs of severe disease^26^. The current study aimed to learn more about canine tick-borne diseases and provides the first sequencing data for *A. marginale* in dogs based on specific genes in southern Egypt, which suggests that dogs can become infected with *A. marginale* and become a source of infection.

## Materials and methods

### Study design, research area and collection of samples

**Figure 1:**
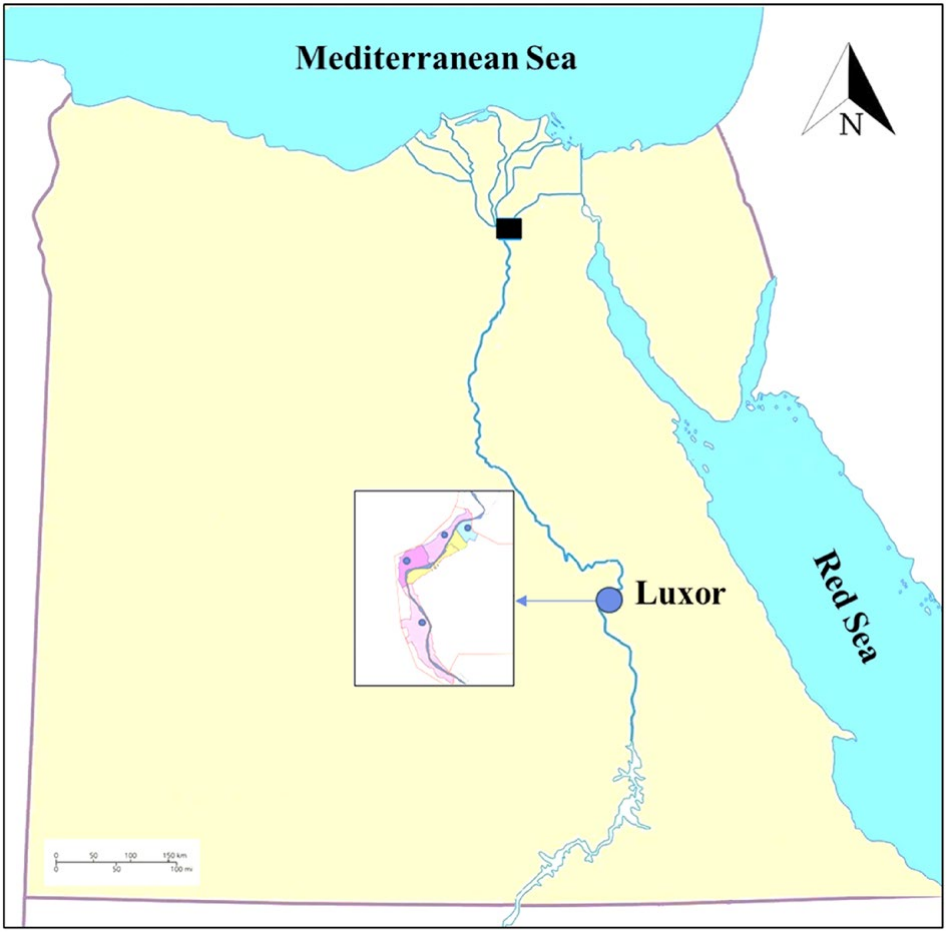
Map of Egypt indicating the study areas where samples were collected from four different regions in Luxor.

Dogs were used in the animal farm for guarding purposes and some dogs were in close contact with the animals especially in the transported animals and during outdoor feeding of the animals. All experiments were performed in accordance with relevant guidelines and regulation rules of the Ethical Research Committee of South Valley University, Faculty of Veterinary Medicine under letter number (No.19/11.08.2021). The current study focuses on the infection of local dog breeds with *A. marginale* and *B. canis vogeli* at various age and sex in the Luxor Governorate. This study occurred from April 2021 to January 2022 (Fig. 1). A total of 100 animals were used in this study, of which 55 were male and 45 were female. The animals ranged in age from one to three years and six months, with the numbers for each age group (1–2-years, 2–3 years, and 3–3.5 years) being 28, 37, and 35, respectively. Using clean, sterile vacutainer tubes with heparin as a target for PCR amplification, whole blood samples were collected from the cephalic veins of the animals for hematological analysis. DNA samples were stored at − 20 °C until use. Availability of samples primarily based on the owner’s cooperation and sampling accessibility at our tested locations determined the current sample numbers, populations, and groupings.

### Clinical and hematological examination of animals

Clinical evaluations of the animals were done, before collecting the samples, for age, with the animals ranging from 1 to 3.5 years, and visible mucous membranes, with 10 dogs appearing pale in the mucous membranes, while 25 of the animals had respiratory disorders and diarrhea. However, 65 dogs were tick-infested, and there were no obvious, distinct clinical manifestations that were detected.

The counts of red blood cells and white blood cells were determined using a (Neubauer hemocytometer, Germany) according to the manufacturer's instructions. A colorimetric endpoint cyanmethemoglobin was used to measure hemoglobin concentration, and packed cell volume was estimated using the microhematocrit centrifugation methods^27^.

### Tick collection and pulverization

The tick samples were carefully cleaned by passing them through a fine mesh and a gentle stream of tap water to get rid of any surface dirt or debris. The ticks were then rinsed twice in sterile Milli-Q water and 70% ethanol for two minutes before being dried to show their taxonomic features. By using the appropriate standard identification keys^28,29^. After that, each tick was carefully put into a 2 ml tube that had a stainless-steel bead affixed to it to make crushing the tick easier after it was frozen for 12 h at − 80 °C. After that, each tick was crushed using an Automill crusher (Tokken. Inc., Chiba, Japan) for three cycles of 30 s each at 2000 rpm. Next, 200 μl of 1 M Tris–HCl (pH 7.5) was added to each sample, and the tubes were centrifuged at 13,000 rpm for 5 minutes at 4 °C. From the resulting mixture, 200 μl of tick homogenate was carefully collected for conventional PCR assay for *mt-rrs* gene detection for tick species identification^30^. Tick 16S rRNA forward and reverse primers used were respectively (5ʹCTGCTCAATGATTTTTTAAATTGCTGTGG3ʹ) and (5ʹCCGGTCTGAACTCAGA TCAAGTA3ʹ) for tick identification, and the PCR conditions are (95 °C)/(5 min) → [(98 °C)/(30 s)–(56 °C)/(30 s)–(72 °C)/(30 s)]30 ×  → (72 °C)/(5 min).

### DNA extraction, detection of control genes and pathogens by PCR

The DNA of one hundred blood samples from dogs was extracted using a (Wizard Genomic DNA Purification Kit, USA) and subjected to conventional Polymerase Chain Reaction (PCR), with the forward and reverse primers used were respectively, (5ʹ GTGAACCTTATCACTTAAAGG 3') and (5ʹ CAACTCCTCCACGCAATCG 3ʹ) for the detection of *small subunit ribosomal RNA* (SSU rRNA) gene of *B. canis vogeli*^31^ and (5ʹ GGGAGCTCCTATGAATTACAGAGAATTGTT 3ʹ), and (5ʹ CCGGATCCTTAGCTGAACAGGAATCTTGC 3ʹ) for the detection of *major surface protein 4* (*msp4*) gene of *A. marginale*^32^. The PCR conditions are (94 °C)/(5 min) → [(94 °C)/(30 s)–(60 °C)/(30 s)–(72 °C)/1 min]35 ×  → (72 °C)/(5 min) for *Babesia canis vogeli* and (94 °C)/(5 min) → [(94 °C)/(30 s)–(60 °C)/(30 s)–(68 °C)/1 min] 35 ×  → (68 °C)/(7 min) for *A. marginale*.

The PCR reaction was done in a total volume of 10 µl using of 5 µl of 2 × Gflex PCR buffer and 0.5 µl of Tks Gflex DNA polymerase (TaKaRa, Shiga, Japan), and 0.5 µl of each of the forward and reverse primers with a concentration of 10 µM, 3 µl of nuclease-free water, and 0.5 µl of the template (DNA template with a concentration ranging from 10 to 30 ng/*µl*).Negative controls containing nuclease-free water were used as negative samples. Electrophoresis of PCR products was done in 1.5% agarose gel in a 1 × Tris–acetate-EDTA (TAE) buffer with an electrophoresis device (Mupid Co., Ltd., Tokyo, Japan). Bands were visualized through a gel documentation system UV device WUV-M20 (ATTO Co., Ltd., Tokyo, Japan) after being stained with 5 μg/ml ethidium bromide in 1 × TAE.

### Sequence and data analysis

Sequence analysis for *A. marginale*, *msp4* genes, and *SSU rRNA* gene of *B. canis vogeli* were subjected to PCR at 50 μl mixtures. The amplicons were purified using NucleoSpin Gel and a PCR Clean-Up Kit (Macherey–Nagel, Leicestershire, Germany) according to the manufacturer's protocol. Sequence readings were compared to the sequences of reported isolates using GenBank. A maximum likelihood phylogenetic tree was constructed using molecular evolutionary genetics analysis, version X (MEGA X software, https://www.megasoftware.net/citations)^33^ with bootstrap values estimated using 1000 replicates based on Kimura model^34^. The 95% confidence intervals were calculated with www.vassarstats.net^35^.

### Ethics statement

The Ethical Research Committee of South Valley University, Faculty of Veterinary Medicine, approved the study (No.19/11.08.2021).

## Result

The number of dogs that had been infected with ticks was 65 out of 100 dogs. The tick infestation generally is different from one or two in dogs, but in some cases the maximum infestation of dogs was 10 ticks per animal, found in the animal ears and groin region. The diseased dogs (25/100) showed diarrhea and cough, and some dogs had poor body conditions (10/100) resembling the emaciation of animals. All dogs with external parasite ticks (65/100) and PCR positive had a pale in the mucous membrane.

Blood samples from animals showed abnormal findings in the blood smear, but the specific type of infection couldn't be identified. Moving forward with molecular examination is a logical step to further investigate and pinpoint the cause of these abnormalities (Supplementary Fig. S1). The results of microscopic examination revealed the presence of two common species of ticks, brown dog ticks *Rhipicephalus sanguineus*, and *Rhipicephalus annulatus* (Supplementary Fig. S2).

**Figure 2:**
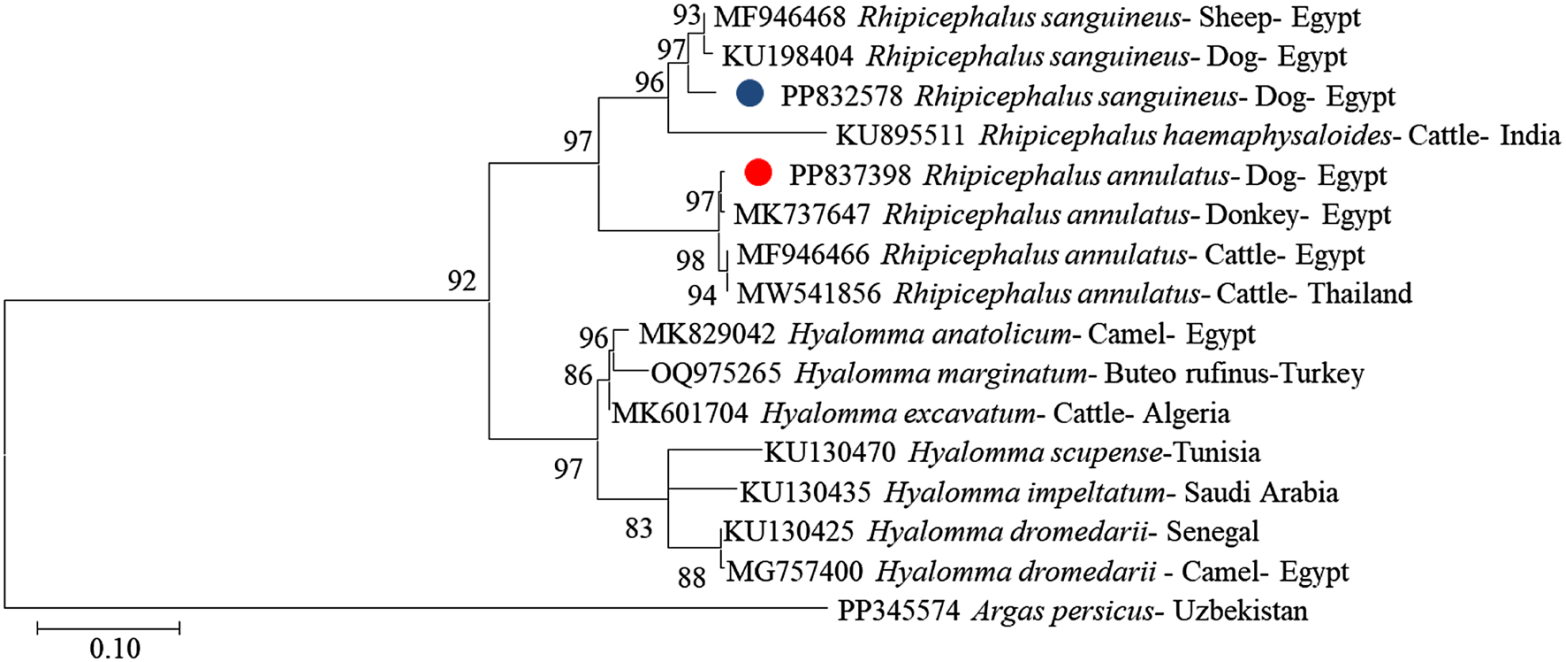
Phylogenetic relations of tick species, obtained via the maximum-likelihood method 606 and the Tamura 3-parameter model based on 16S rRNA gene sequences. The percentage trees in which the related taxa clustered together is displayed next to the branches. Branch 608 lengths are expressed in terms of the number of substitutions per site, and the tree is drawn to scale. The circles represent the tick species infesting dogs in the present study.

The microscopic results were confirmed by sequencing and phylogenetic analysis, with our isolate of *Rhipicephalus annulatus* (PP837398.1) exhibiting 99.76% identity alignment when compared to amplicons that are distinct from previously published isolates in GenBank MK737647.1, and *Rhipicephalus sanguineus* PP832578.1 were identified with previously published isolates in GenBank MW560390.1 by 99.75% (Fig. 2).

Molecular identification for the tick species and *A. marginale msp4* gene and *B. canis vogeli SSU rRNA* gene by PCR reaction was shown in (Supplementary Figs. S3 and S4). The results of infection rates for *A. marginale* and *B. canis vogeli* and hematological parameters are shown in Tables 1 and 2. Eight out of a total of one hundred animals (8%) tested positive for *A. marginale* and *B. canis vogeli*. Additionally, male animals (75%) had a greater infection rate than female animals (25%), with most of the infections occurring in the northern part of the governorate (75%). The total number of white blood cells and eosinophils increased slightly, and total red blood cells and hemoglobin decreased more than the reference parameter, with all other hematological parameters remaining within the normal range (Table 1). The results showed that *A. marginale* and *B. canis vogeli* had equal infection rates (4%) (Table 2). In addition to the location, the highest infection rate of *A. marginale* was found in the northern (50%), middle (25%), and southern areas (25%) of Luxor governorate, respectively. Regarding age, the highest infection rate (75%) was found in older animals (older than one year) than in younger (less than one year) animals (25%).

**Table 1:** Hematological parameters of dogs.

Parameters	Mean ± SE	Reference range
RBCs (10^6^ cells/µl)	5.40 ± 0.23	5.5–8.5
WBC (10^3^ cells/µl)	17.17 ± 0.91	5.9–16.6
HB (g/dl)	12.79 ± 3.24	14.2–19.2
PCV (%)	41.48 ± 1.70	29–55
MCV(fl)	79.76 ± 3.6	65–80
MCH (pg)	18.38 ± 0.95	12.2–25.4
MCHC (%)	23.23 ± 0.78	32–36
Neutrophils (%)	54.51 ± 2.39	51–84
Lymphocyte (%)	32.45 ± 1.23	8–38
Monocyte (%)	2.03 ± 0.27	1–9
Eosinophils (%)	9.33 ± 1.10	0–9
Basophil (%)	0.51 ± 0.09	0–1

**Table 2:** *Anaplasma marginale* and *Babesia canis vogeli* in dogs based on PCR detection in blood samples.

Parasite	Number of animals	Number of negative	Number of positive	Percent positive	95% CI
*Anaplasma marginale*	100	96	4	4.0%	1.3–10.5
*Babesia canis vogeli*	100	96	4	4.0%	1.3–10.5
Total	100	92	8	8.0%	3.8–15.6

95% CI calculated according to the method described at http://vassarstats.net/.

*CI* confidence interval.

**Figure 3:**
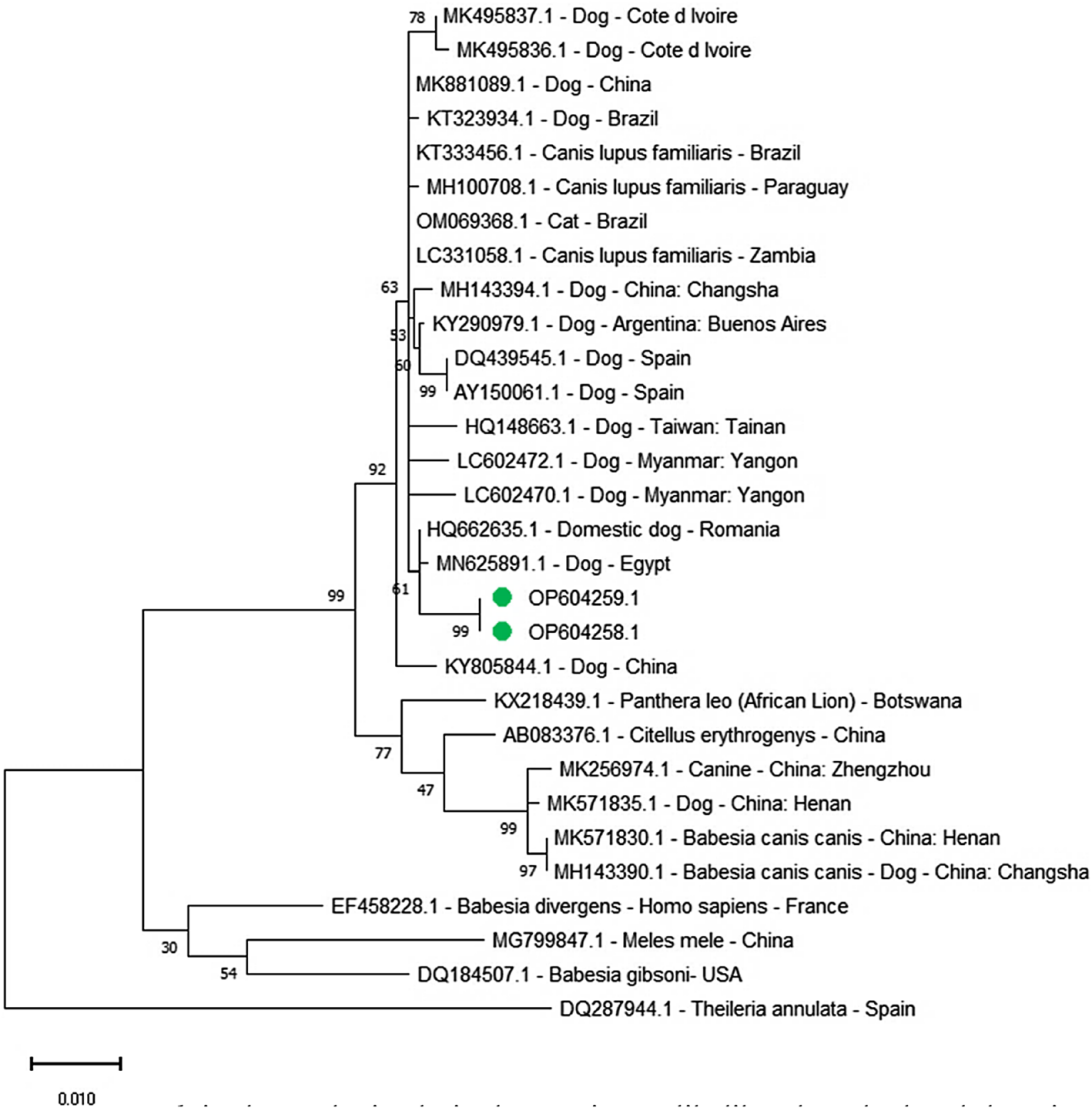
Phylogenetic relations of *Babesia canis vogeli* in dogs, obtained via the maximum-likelihood method and the Kimura two-parameter model based on small subunit ribosomal RNA gene sequences. The percentage of trees in which the related taxa clustered together is displayed next to the branches. Branch lengths are expressed in terms of the number of substitutions per site, and the tree is drawn to scale. The circles represent *Babesia canis vogeli* in dogs obtained in the present study.

The phylogenetic analysis for the *B. canis vogeli SSU rRNA* gene in this study OP604258.1 and OP604259.1 compared with amplicons separate from other reported isolates with alignment by identity by 99.89% MN625891.1 with dog from Egypt, and by 99.78% HQ662635.1 with dog from Romania, and by 99.74% MH100708.1 and OM069368.1 with *Canis lupus familiaris* from Paraguay and cat from China and by 99.66% LC331058.1 with *Canis lupus familiaris* from Zambia and by 99.61% KT323934.1 with dog from Brazil, and by 99.55% MK881089.1 and KT333456.1 with dog from China and *Canis lupus familiaris* from Brazil, and by 99.33% KY290979.1 with dog from Argentina and by 99.32% MH143394.1 with dog from China and by 99.30% MK495837.1 and MK495836.1 with dog from Cote d Ivoire and by 98.99% HQ148663.1 and Spain and by 98.88% KY805844.1 with dog from China and by 98.92% AY150061.1 with dog from Spain and by 98.32% LC602470.1 with dog from Myanmar, and by 97.96% AB083376.1 with *Citellus erythrogenys* from China and by 97.43% KX218439.1 with *Panthera leo* (African Lion) from Botswana, and by 97.39% MK571835.1 with dog from China, and by 97.32% MK256974.1 with canine form China, and by 97.28% MH143390.1 with dog from China and by 97.03% LC602472.1 with dog from Myanmar (Fig. 3).

**Figure 4:**
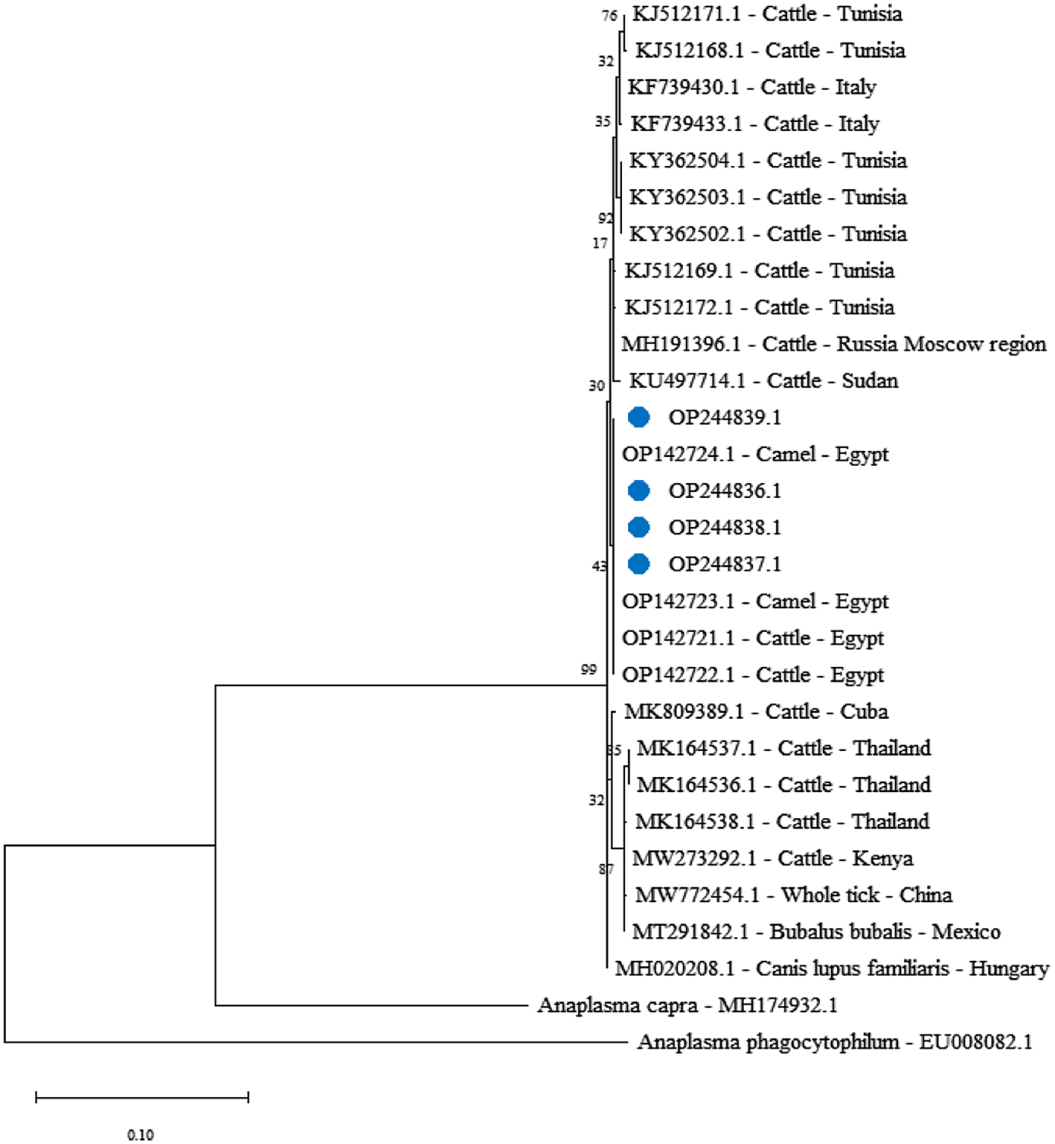
Phylogenetic relations of *Anaplasma marginale* obtained via the maximum-likelihood method and the Kimura two-parameter model based on *major surface protein 4* (*msp4*) gene sequences. The percentage of trees in which the related taxa clustered together is displayed next to the branches. Branch lengths are expressed in terms of the number of substitutions per site, and the tree is drawn to scale. The circles represent *Anaplasma marginale* obtained in the present study.

The *A. marginale* (*msp4*) gene was sequenced for phylogenetic analysis and genotyping of *A. marginale* in dogs from different locations in the Luxor Governorate in southern Egypt. All sequences were also submitted to GenBank, and the following accession numbers can be used to access them for the *msp4* gene OP244836.1, OP244837.1, OP244838.1, and OP244839.1. The phylogenetic analysis for the *msp4* gene in this study was compared with amplicons separate from other reported isolates with alignment by identity 100% with cattle and camels from Egypt OP142721.1, OP142722.1, OP142723.1, and OP142724.1; by identity 99.72% with *Canis lupus familiaris* from Hungary MH020208.1, cattle from Russia MH191396.1, cattle from Tunisia KJ512169.1, KJ512172.1; by identity 99.57% with cattle from Tunisia KJ512168.1, KJ512171.1, cattle from Sudan KU497714.1; by identity 99.43% with cattle from Italy KF739430.1, KF739433.1, and Cuba MK809389.1; 99.29% with cattle from Tunisia KY362502.1, KY362503.1, KY362504.1; by identity 99.15% with whole ticks from China MW772454.1, cattle from Thailand MK164536.1, MK164537.1, MK164538.1, *Bubalus bubalis* from Mexico MT291842.1, cattle from Kenya MW273292.1 (Fig. 4).

## Discussion

In Egypt, dogs have recently received much attention from scientists, particularly in the southern region, and many findings have begun to be published. A simple method that is commonly used in clinical diagnosis is the microscopic examination of stained thin blood smears^36^. However, microscopic examination lacks sensitivity and makes it challenging to distinguish between species with mixed infections and low parasitemia. In addition to personal experience in evaluating blood smears, the diagnosis is frequently inaccurate^37^. The polymerase chain reaction assay, a variable amplification method, has exceptionally high sensitivity and is often used for research purposes^38^. For this reason, in our study, we focused on molecular techniques for the diagnosis and characterization of tick-borne disease.

The clinical and hematological examination of dogs in this study revealed no specific clinical signs for babesiosis and only a slight increase in total white blood cell counts and eosinophils, which could be related to tick infestation. These findings are consistent with the available literature, which regards *B. vogeli* as the lowest pathogenic strain, causing a relatively mild disease, usually without evidence of clinical signs, and can be found in clinically healthy dogs^39^.

Microscopic examination of ticks revealed the presence of two types of ticks in dogs, *Rhipicephalus sanguineus*, which primarily infects dogs, and *Rhipicephalus annulatus*, which primarily infects cattle as well as some domestic animals like horses and dogs^40^. The phylogenetic analysis of our isolate, *Rhipicephalus annulatus* (PP837398.1), exhibited 99.76% identity alignment compared to other amplicons in GenBank MK737647.1, and *Rhipicephalus sanguineus* (PP832578.1) was identified with MW560390.1 by 99.75%. The mitochondrial *16S rRNA* gene serves as a valuable marker for identifying ticks^41^.

This study is focused on tick-borne diseases in dogs, which include *Babesia* and *Anaplasma*. *Babesia canis vogeli* is the most widely distributed *Babesia* subspecies, occurring in Africa^42,43^, Europe^44,45^, Asia^46,47^, and Australia^48,49^. *Babesia canis vogeli* has been reported in Colombia^50^, Venezuela^51^, Brazil^52^, and Argentina^53^. In this study, the infection rate of *B. canis vogeli* in dogs from southern Egypt was 4%, which was lower than the result in dogs from northern Egypt, where the occurrence of *B. canis vogeli* infection was 5.1%^54^. We found that our reported rate of *B. canis vogeli* was lower than that in Iraq (5.1%)^55^, Brazil (4.8% and 15.6%)^56^, Iran (9.3%), Bosnia (85%), and Nigeria (10.8%)^57–59^. In this study, all dogs were local breeds, and babesiosis is more common and severe in imported dogs than in native species. However, not all dog breeds are equally susceptible to babesiosis^60^.

The current findings related to sex confirm that males have a higher probability of being infected with *B. canis vogeli* (75%) than females, only one animal was positive (25%) of being infected with *B. canis vogeli*. This result agreed with previous reports that males were more susceptible than females to *Babesia* infection^61^. The results of phylogenetic analysis for *B. canis vogeli* were obtained by comparing the *B. canis vogeli* sequences of Egypt isolates and other registered sequences in the gene bank. The phylogenetic analysis for the *SSU rRNA* gene was compared with amplicons isolated from other reported isolates, with alignment by identity ranging from 99.89% with accession number MN625891.1 to 97.03% with accession number LC602472.1. Two *B. canis vogeli* vouchers were clustered in distinct lineages and shared a well-supported subclade with parasites of the same host (Fig. 3).

Our study is the second report on *A. marginale* in dog blood in Egypt and the first report in southern Egypt in Luxor governorate. The first report was based on *16S ribosomal RNA* sequences of Anaplasmataceae^62,63^. Major surface proteins have been designated as 1a, 1b, 2, 3, 4, and 5^64^, these proteins are recognized by neutralizing antibodies and have a strong intermolecular interaction in the membrane of the initial bodies, where they perform important functions^65^. Anaplasma species' biogeography and evolution have been studied by phylogenetic analysis using the *msp4* gene^66^. Our report is considered the first report in Egypt for *A. marginale* in dogs based on the *msp4* gene, which was sequenced for phylogenetic analysis.

All sequences were submitted to GenBank with accession numbers OP244836.1, OP244837.1, OP244838.1, and OP244839.1. Based on previous GenBank data, it was aligned by identity 100% with cattle and camels from Egypt, and by identity 99.72% with *Canis lupus familiaris* from Hungary, cattle from Russia, cattle from Tunisia, and by identity 99.57% with cattle from Tunisia and Sudan, and by identity 99.43% with cattle from Italy. It showed our sequences of *A. marginale* clustered together and shared a well-supported subclade with parasites of the same species (Fig. 4).

Based on the sequence data, the construction of the phylogenetic tree demonstrated the splitting of the tree into two major clades, and the phylogenetic position of the nucleotide sequences of *A. marginale* identified here was clustered with other *A. marginale* infecting other animals. The similarity between the sequences isolated from dogs and those isolated from cattle and camels in southern Egypt may be reflected in the close contact between dogs and these animals in rural areas. In an unpublished report, *A. marginale* was found in dogs in Hungary and was registered in GenBank based on the 16S ribosomal RNA gene under accession number MH020201. This finding supports the idea that some tick-borne pathogens accidentally parasitize other hosts^67^.

Based on the obtained data in the current study, future studies will be conducted including a higher number of samples from different Egyptian regions and more factors will be tested to identify the risk of infections. In this regard, different approaches will be additionally implemented such as serological testing and tick examinations.

## Conclusions

The findings of this study revealed the widespread and considerable prevalence of *A. marginale* and *B. canis vogeli* in dogs in Luxor, Egypt. The identity of *A. marginale* was confirmed by amplifying the specific *msp4* gene, which provided new data on *A. marginale* in dogs in southern Egypt. The phylogenetic analysis for the *SSU rRNA* gene of *B. canis vogeli* did not show 100% identity with any previously deposited sequence in GenBank (the maximum identity was 99.89%). The molecular characterization of *A. marginale and B. canis vogeli* in dogs is considered the first report in this area and provides significant data to the veterinary and public health sectors.

### Supplementary Information

Supplementary Figures.

## Data Availability

Sequence data that support the findings of this study have been deposited in GenBank with the accession numbers: OP244836.1, OP244837.1, OP244838.1, OP244839.1, OP604258.1, OP604259.1, PP832578.1, and PP837398.1
