# Supplementary material for: Molecular detection and characterization of Anaplasma marginale and Babesia canis vogeli infecting dogs in Luxor, Egypt
Source: Sci Rep. 2024 Jul 23;14:16888. doi: 10.1038/s41598-024-67009-6 (PMC11266501; doi:10.1038/s41598-024-67009-6)
Supplement: Supplementary file 1 — Supplementary Figures. [file 41598_2024_67009_MOESM1_ESM.pptx]

## Slide 1
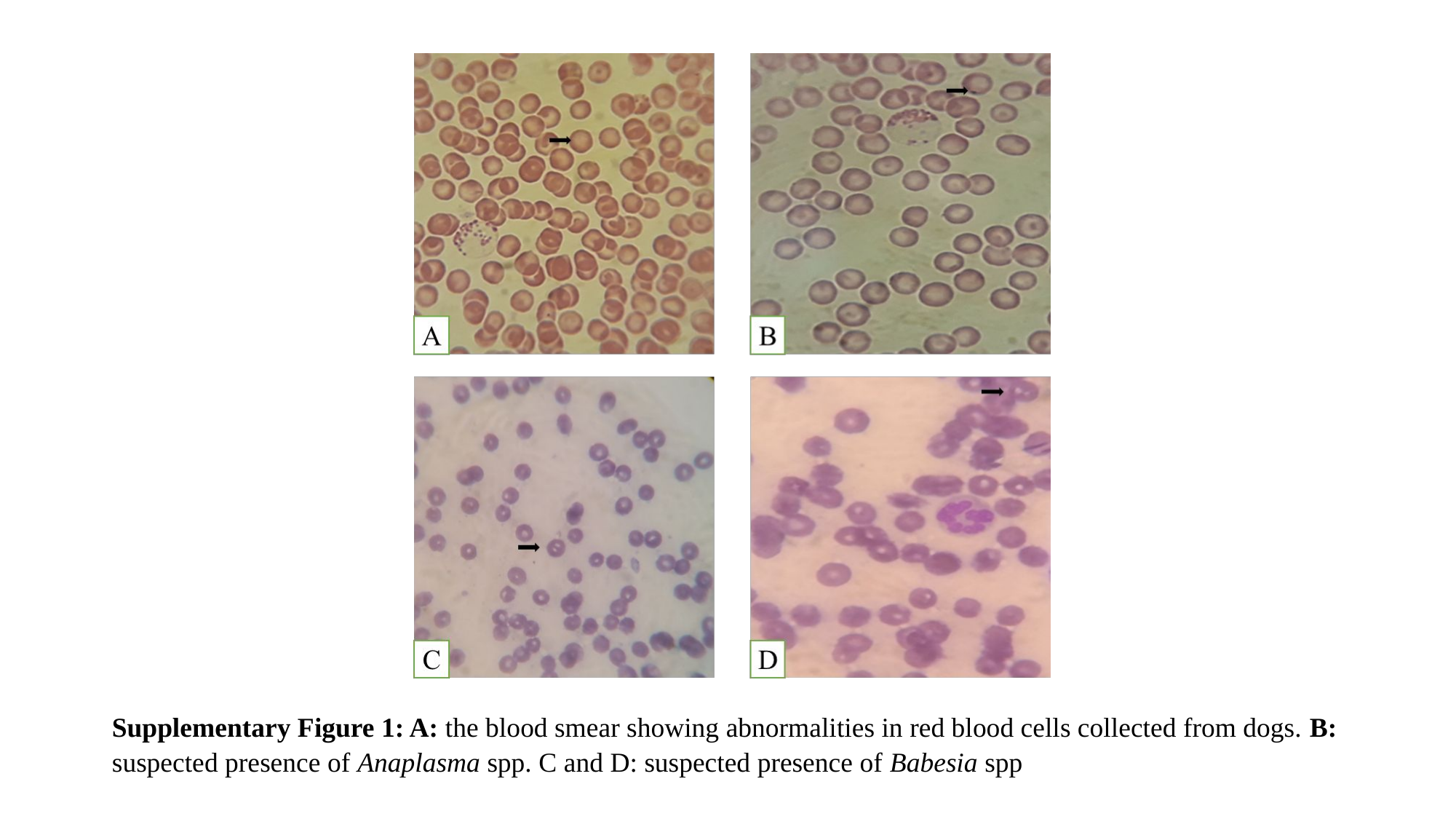

Supplementary Figure 1: A: the blood smear showing abnormalities in red blood cells collected from dogs. B: suspected presence of Anaplasma spp. C and D: suspected presence of Babesia spp

## Slide 2
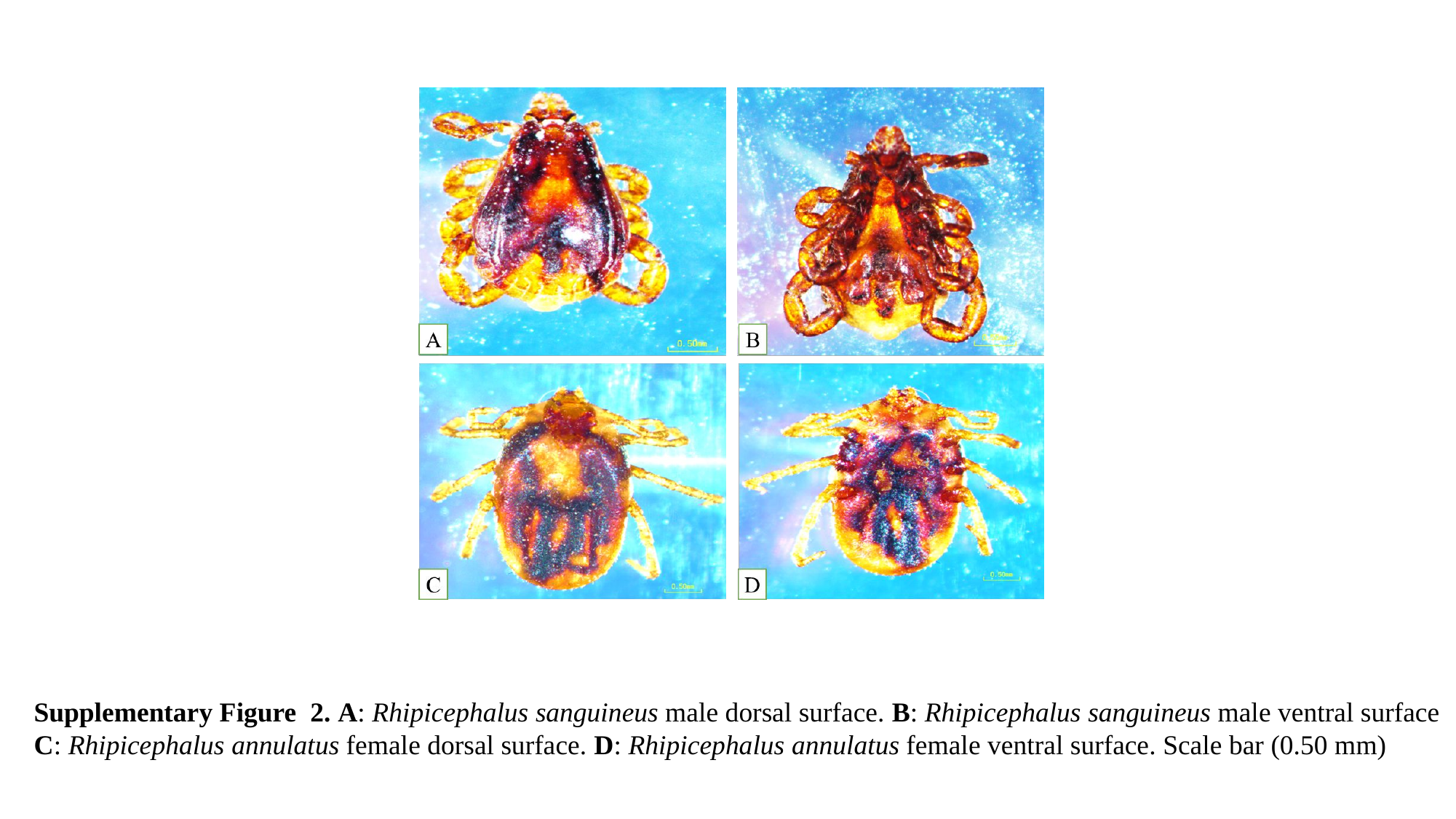

Supplementary Figure 2. A: Rhipicephalus sanguineus male dorsal surface. B: Rhipicephalus sanguineus male ventral surface
C: Rhipicephalus annulatus female dorsal surface. D: Rhipicephalus annulatus female ventral surface. Scale bar (0.50 mm)

## Slide 3
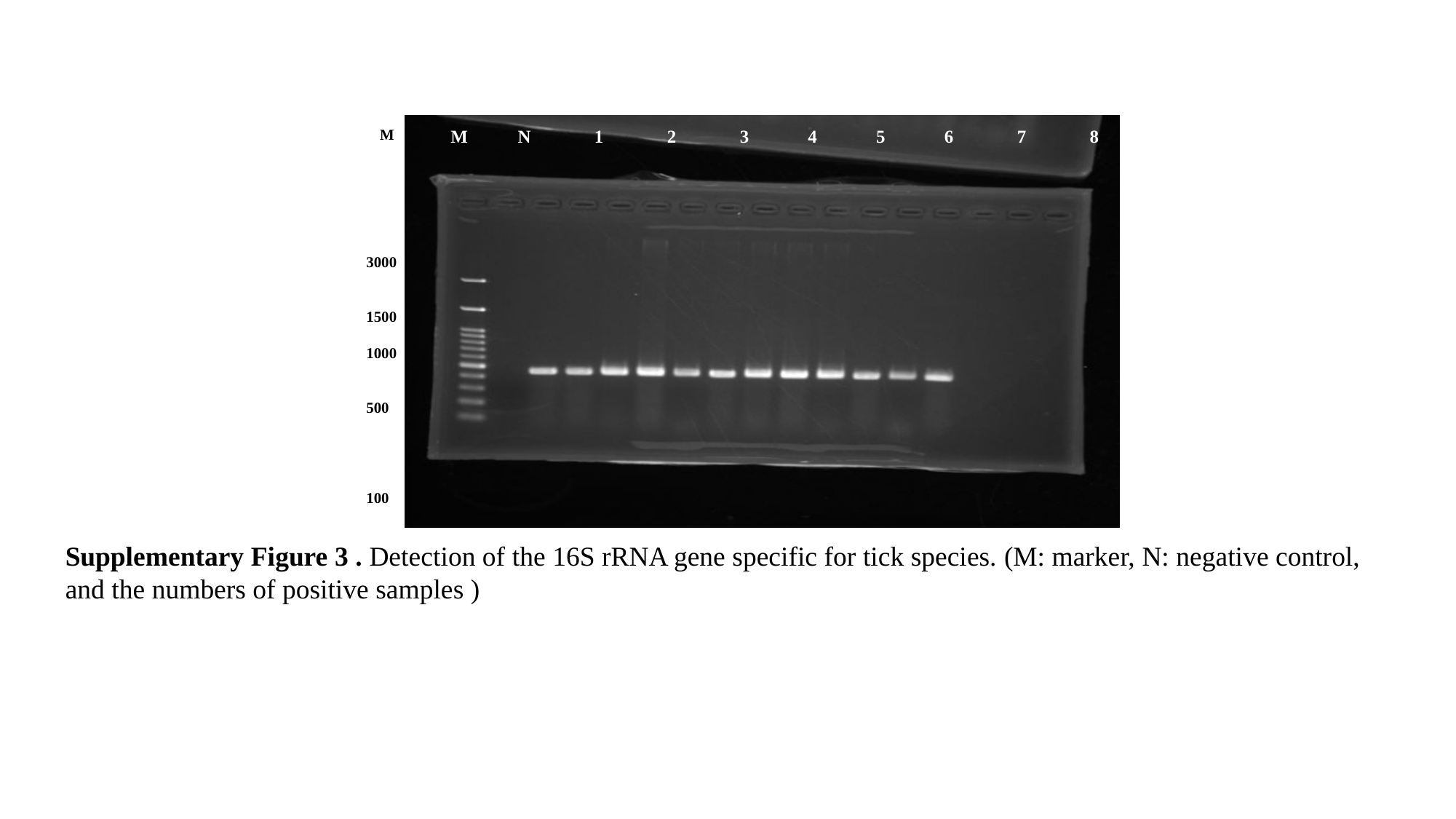

M N 1 2 3 4 5 6 7 8
M
3000
1500
1000
500
100
Supplementary Figure 3 . Detection of the 16S rRNA gene specific for tick species. (M: marker, N: negative control, and the numbers of positive samples )

## Slide 4
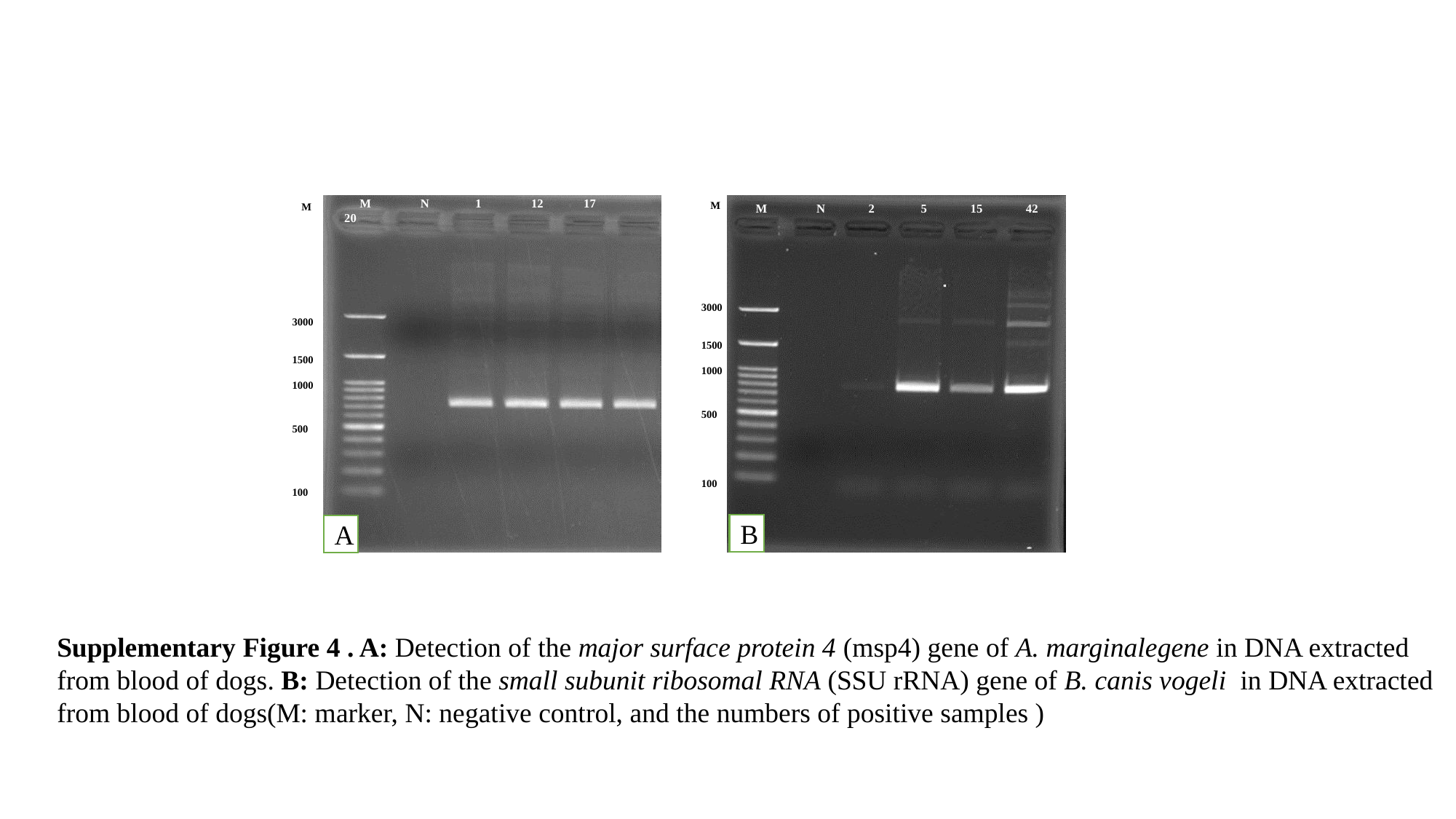

M
3000
1500
1000
500
100
 M N 1 12 17 20
M
3000
1500
1000
500
100
 M N 2 5 15 42
B
A
Supplementary Figure 4 . A: Detection of the major surface protein 4 (msp4) gene of A. marginalegene in DNA extracted from blood of dogs. B: Detection of the small subunit ribosomal RNA (SSU rRNA) gene of B. canis vogeli in DNA extracted from blood of dogs(M: marker, N: negative control, and the numbers of positive samples )
